# Intrathecal B-cell activation in LGI1 antibody encephalitis

**DOI:** 10.1212/NXI.0000000000000669

**Published:** 2020-02-06

**Authors:** Klaus Lehmann-Horn, Sarosh R. Irani, Shengzhi Wang, Arumugam Palanichamy, Sarah Jahn, Ariele L. Greenfield, Ravi Dandekar, Gildas Lepennetier, Sophia Michael, Jeffrey M. Gelfand, Michael D. Geschwind, Michael R. Wilson, Scott S. Zamvil, H.-Christian von Büdingen

**Affiliations:** From the Department of Neurology (K.L.-H., S.W., A.P., S.J., A.L.G., R.D., J.M.G., M.D.G., M.R.W., S.S.Z., H.-C.v.B.), UCSF Weill Institute for Neurosciences; Program in Immunology (K.L.-H., S.S.Z.), UCSF, San Francisco, CA; Department of Neurology (K.L.-H., G.L.), Klinikum rechts der Isar, Technische Universität München, Germany; and Oxford Autoimmune Neurology Group (S.R.I., S.M.), John Radcliffe Hospital, University of Oxford, UK.

## Abstract

**Objective:**

To study intrathecal B-cell activity in leucine-rich, glioma-inactivated 1 (LGI1) antibody encephalitis. In patients with LGI1 antibodies, the lack of CSF lymphocytosis or oligoclonal bands and serum-predominant LGI1 antibodies suggests a peripherally initiated immune response. However, it is unknown whether B cells within the CNS contribute to the ongoing pathogenesis of LGI1 antibody encephalitis.

**Methods:**

Paired CSF and peripheral blood (PB) mononuclear cells were collected from 6 patients with LGI1 antibody encephalitis and 2 patients with other neurologic diseases. Deep B-cell immune repertoire sequencing was performed on immunoglobulin heavy chain transcripts from CSF B cells and sorted PB B-cell subsets. In addition, LGI1 antibody levels were determined in CSF and PB.

**Results:**

Serum LGI1 antibody titers were on average 127-fold higher than CSF LGI1 antibody titers. Yet, deep B-cell repertoire analysis demonstrated a restricted CSF repertoire with frequent extensive clusters of clonally related B cells connected to mature PB B cells. These clusters showed intensive mutational activity of CSF B cells, providing strong evidence for an independent CNS-based antigen-driven response in patients with LGI1 antibody encephalitis but not in controls.

**Conclusions:**

Our results demonstrate that intrathecal immunoglobulin repertoire expansion is a feature of LGI1 antibody encephalitis and suggests a need for CNS-penetrant therapies.

Leucine-rich, glioma-inactivated 1 (LGI1) antibody encephalitis is characterized by rapidly progressive cognitive impairment, frequent seizures, most characteristically faciobrachial dystonic seizures, psychiatric disturbances, and sleep alterations.^1,2^ These distinctive clinical features, alongside in vitro and in vivo studies,^3,4^ and the often rapid response of seizures to immunotherapies all strongly suggest that LGI1 antibodies are pathogenic.^2^ However, LGI1 antibody encephalitis can often result in residual cognitive impairment and neurologic disability: this represents an unmet medical need.^2,5^

Although CSF LGI1 antibodies are detected in around 90% of cases, this condition is infrequently associated with CSF lymphocytosis or oligoclonal bands.^2,6,7^ Therefore, the CSF B-cell response has received limited consideration as contributor to pathogenesis or as a potential therapeutic target. Indeed, very little is known about B cells that participate in the autoimmune response against LGI1, either in the periphery or CSF. Here, we applied deep B-cell immune repertoire sequencing (DIRS) on sorted peripheral blood (PB) B-cell subsets and CSF and found strong evidence for intrathecal antigen-driven immune responses in patients with LGI1 antibody encephalitis. These observations inform disease biology and suggest CNS B cells as a candidate therapeutic target in these patients.

## Methods

### Patient samples

Six patients with LGI1 antibody encephalitis from the University of California, San Francisco (UCSF) Autoimmune Encephalopathy Clinic underwent collection of paired PB (40 mL) and 10–25 mL of CSF. B-cell subsets were isolated as described previously.^8^ As controls, 2 patients with other noninflammatory neurologic diseases from the same center were included in the study and their PB and CSF samples collected accordingly.

### Standard protocol approvals, registrations, and patient consents

The study was approved by the Institutional Review Board of the UCSF. Written informed consent was obtained from all participants in the study.

### Cell staining and sorting

Ficoll-density separated peripheral blood mononuclear cells were stained with the following antibodies: CD19 (APC Cy7), immunoglobulin D (IgD) (PE Cy7), CD27 (Qdot605), CD38 (PerCP Cy5.5), and CD3 (Pacific blue) as previously described.^8^ B-cell subsets were sorted using a FACS Aria III (BD Biosciences, Franklin Lakes, NJ) into naive (CD19^+^IgD^+^CD27^−^), unswitched memory (CD19^+^IgD^+^CD27^+^), switched memory (CD19^+^IgD^−^CD27^+^CD38^−^), double negative (CD19^+^IgD^−^CD27^−^), and plasmablasts/plasma cells (CD19^+^IgD^−^CD27^hi^CD38^hi^). Sorted cells were immediately lyzed in RLT buffer (RNeasy kit; Qiagen, Hilden, Germany) and stored at −80°C. To preserve the far lower CSF lymphocyte frequencies, unfractionated pelleted CSF cells were studied.

### Immunoglobulin messenger RNA amplification and immunoglobulin repertoire sequencing

Sequencing work flow was performed as previously described,^9^ with modifications to sequence human samples. In brief, total RNA was isolated from CSF cells and PB B-cell subsets, followed by reverse transcription into complementary DNA (cDNA). Next, immunoglobulin G (IgG) heavy chain variable region (VH) and immunoglobulin M (IgM) VH were amplified by PCR using the following primers: IgG 3′ primer: 5′-GGGAAGACSGATGGGCCCTTGGTGG-3′; IgM 3′ primer: 5′-GCTCGTATCCGACGGG-3′; an equimolar mix of 7 VH family 5′ primers: VH1: 5′-GAARRTYTCCTGCAAGGYWTC-3′; VH2: 5′-CACRCTGACCTGCACCKTCTC-3′; VH3: 5′-KARACTCTCCTGTRCAGCCTB-3′; VH4: 5′-GTCCCTCACCTGCRCTGTCTM-3′; VH5: 5′-GARGATCTCCTGTAAGGGTTC-3′; VH6: 5′-CTCACTCACCTGTGCCATCTC-3′; VH7: 5′-GAAGGTTTCCTGCAAGGCTTC-3′. PCR conditions were (1) 95°C, 60 seconds; (2) 95°C, 30 seconds; 66.6°C, 30 seconds; 72°C, 60 seconds (33 or 45 cycles); and (3) 72°C, 7 minutes. Specific PCR products were gel purified and mixed to create 15 pM cDNA libraries, which were analyzed by Ion Torrent semiconductor sequencing.

### Sequence analysis

IGHV and IGHJ gene segment usage, complementarity determining region (CDR)1-3 amino acid sequence, and number of somatic hypermutation (SHM) events were determined as previously described.^8,9^ Briefly, CDR3 amino acid sequences were determined using a custom-made pipeline adapted from the AbMining tool,^10^ and identified CDR3 regions were related to IGHV and IGHJ germline genes using IgBlast. To calculate SHM profiles, sequencing reads with identical CDR1 to CDR3 nucleotide sequences were grouped as nonredundant (unique) reads, and SHMs were quantified for this entire region based on the alignment of reads with germline gene segments.

Compartmental connectivity via bicompartmental clustering of IgM-VH or IgG-VH from CSF and PB B-cell subsets was performed as previously described.^8^ Briefly, clonally related Ig-VH sequences were identified based on H-CDR3 similarity (hamming distance of H-CDR3 amino acid sequence less than 2) and usage of identical Ig germline segments. For lineage analysis, only Ig-VH sequences with in-frame H-CDR3 and which spanned at least from the 5′ end of H-CDR1 to the 3′ end of H-CDR3 with a contiguous reading frame were used; IgTree^11^ (kindly provided by Dr. Ramit Mehr, Bar-Ilan University, Ramat-Gan, Israel) was used to map the lineage.^8^ Putative germline nodes are inferred, and lineage intermediates not found by DIRS were calculated by IgTree.

### Data availability

All next-generation sequencing data and computer code other than software packages are available from the corresponding author on reasonable request.

## Results

**Figure 1:**
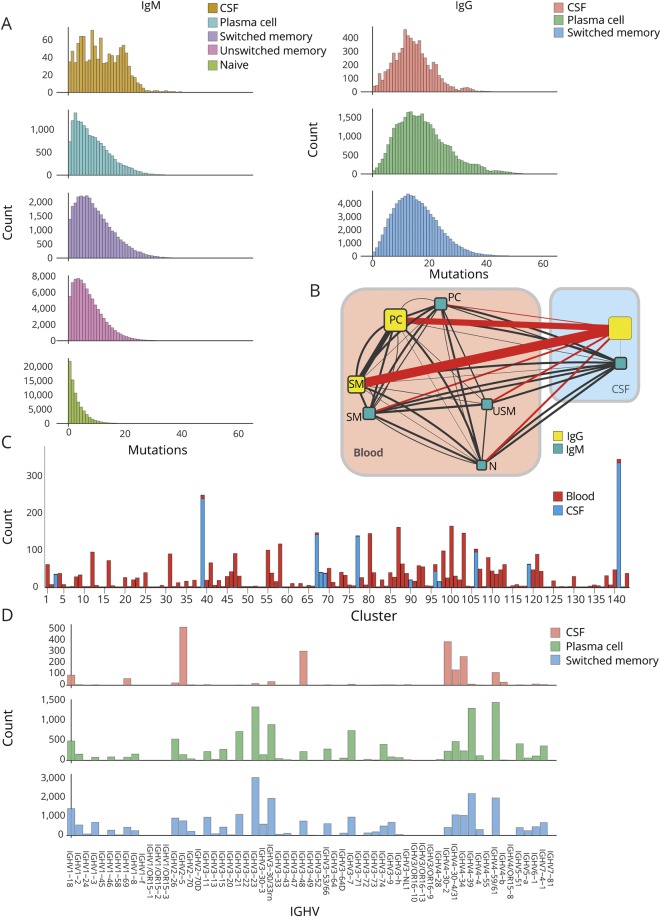
In patients with leucine-rich, glioma-inactivated 1 antibody encephalitis, deep immune repertoire sequencing of the immunoglobulin heavy chain variable region (Ig-VH) shows connectivity of class-switched mature peripheral blood (PB) B cells to clonally expanded CSF B cells/clusters One representative patient of 6 patients is shown in A–D. (A) Cumulative number of somatic mutations (x-axis) in the IGHV gene nucleic acid sequence excluding the CDR3 region in nonredundant sequences (count; y-axis) of sorted PB B-cell subsets and CSF B cells are shown for IgM (left panels) and IgG (right panels). (B) Connectivity of the various PB and CSF B-cell compartments based on clonal relationship is illustrated. While the size of the boxes correlates with the number of reads in each compartment, the thickness of the connecting line correlates with the number of related sequences between the 2 compartments it connects. For better display, all lines connecting to the CSF IgG compartment are depicted in red, whereas all others are in black. IgG = immunoglobulin G; IgM = immunoglobulin M; N = naive; PC = plasmablast/plasma cell; SM = switched memory; USM = unswitched memory. (C) All clusters of clonally related Ig-VH that have at least 1 sequence in one of the PB compartments and 1 in the CSF compartments (shared PB and CSF clusters) are depicted with their respective size (number of nonredundant sequences) on the y-axis. (D) Frequency of used IGHV genes in the IgG CSF, PB plasmablast/plasma cell, and PB SM cell subsets.

Serum LGI1 antibodies titers were on average 127-fold higher than CSF LGI1 antibody titers by live cell–based assay using a membrane-tethered LGI1 construct in all 6 patients with LGI1 antibody encephalitis (table), none of whom were asymptomatic at follow-up.^12^ None of the 6 patients showed a CSF lymphocytosis or oligoclonal bands on routine CSF analysis. There was no abnormal enhancement on brain MRI in any of the patients following the administration of gadolinium, consistent with no substantial blood-brain barrier (BBB) opening. DIRS of IgG-VH and IgM-VH was performed from paired CSF and B-cell subsets sorted from peripheral blood mononuclear cell samples of the 6 LGI1 antibody encephalitis patients, and a median of 600,707 sequences per sample (range 165,369–1,586,974) were generated. As expected, circulating naive B cells had limited somatic mutations and as cells acquired the postgerminal center marker CD27, and class-switched to IgG, mutations accumulated (figure 1A). These findings are a validation that DIRS reliably reflects the conventional B-cell maturation stages and suggests that CSF B cells in LGI1 encephalitis have undergone antigen-driven maturation (figure 1A).

**Table:**
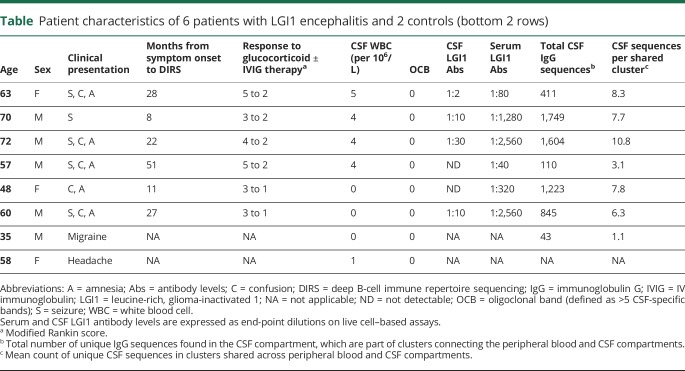
Patient characteristics of 6 patients with LGI1 encephalitis and 2 controls (bottom 2 rows)

Overall, across all 6 patients, all B-cell subsets of IgG and IgM isotypes showed high degrees of sequence connectivity in both PB and CSF compartments (figure 1B). A striking link existed between PB IgG-expressing SM cells/plasmablasts/plasma cells and the IgG-expressing CSF B cells, suggesting that class-switched mature B-cell compartments are consistently connected across the BBB. The CSF B cells often represented a discrete number of highly expanded clusters (figure 1C). Indeed, this restriction of the CSF B-cell repertoire was also evident at the level of the heavy V gene family usage. In contrast to the peripheral SM and plasmablast/plasma cell IgG compartments, which were diverse and highly comparable, the CSF IgG repertoire was distinct and restricted (figure 1D).

**Figure 2:**
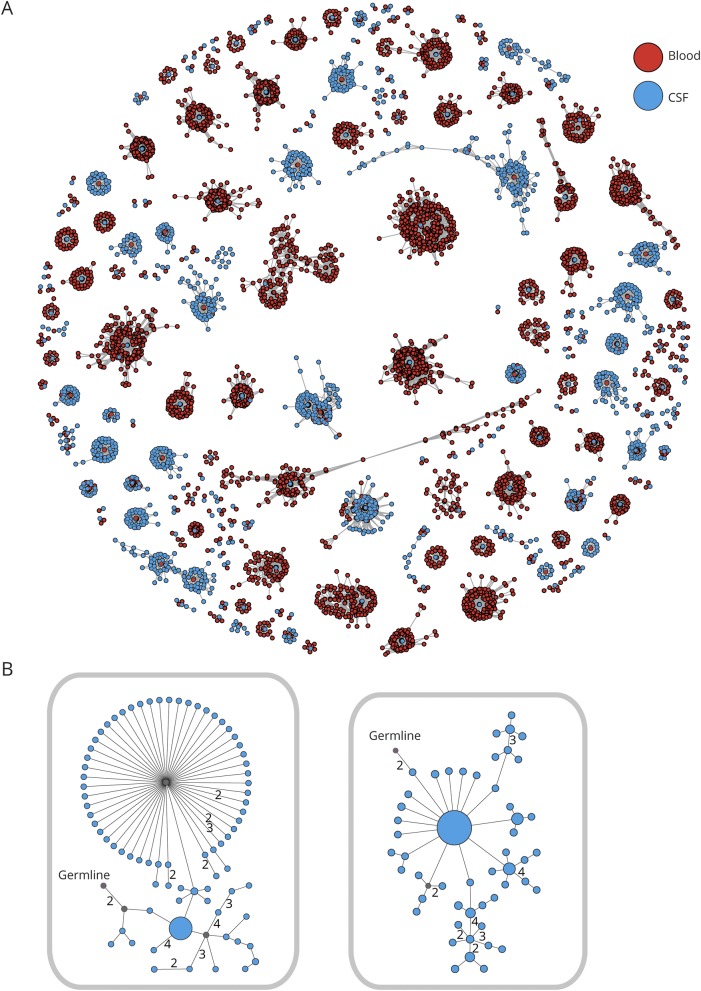
Intrathecal somatic hypermutation in patients with leucine-rich, glioma-inactivated 1 (LGI1) antibody encephalitis (A) Data from 1 representative patient of 6 LGI1 patients showing all clusters of clonally related immunoglobulin heavy chain variable region, which are shared between peripheral blood (PB) and CSF B cells. Each red dot represents ≥1 identical PB sequence, and each blue dot ≥1 identical CSF sequence. Two dots connected by a line differ from each other by a Hamming distance of 1 in their CDR3 region on the nucleotide sequence level. Clusters of related sequences are grouped together. (B) Two Ig lineage trees of CSF B cells from 1 patient are shown. Each dot represents 1 sequence, and its size correlates with the number of times this sequence could be found. Two dots connected by a line differ from each other by 1 nucleotide in the CDR3 region unless marked otherwise. Putative germline nodes are labeled; lineage intermediates not found in the sequencing data were calculated and are labeled in gray.

Next, clusters shared between the CSF and PB were examined more closely. These shared CSF/PB clusters on average had 990.3 (range 110–1,749) unique sequences. In this analysis, each unique sequence was represented by a dot and a line joining neighboring sequences represented a mutational (Hamming) distance of 1 in their CDR3 region (figure 2A). By definition, there is at least 1 PB (red) and 1 CSF (blue) sequence in each cluster. Many shared clusters were dominated by peripheral B cells and were related to only a small number of CSF B cells. Also, of interest, several clusters highly dominated by CSF sequences were apparent and often showed a PB sequence in the center, representing a more proximal sequence with fewer mutations from which surrounding CSF sequences may have descended. More detailed analysis of Ig lineage trees from clonally related CSF IgG-VH demonstrated intensive mutational activity, again suggesting intrathecal SHM (figure 2B).

In contrast to all LGI1 antibody encephalitis cases, 2 patients ultimately diagnosed with noninflammatory neurologic diseases (1 with headache and 1 with chronic migraine), which were analyzed following the same protocol, did not show comparable intrathecal B-cell activity. In 1 of the 2 controls, we were unable to amplify enough CSF IgG RNA for sequencing, indicating very few CSF IgG transcripts; in the other, we were able to detect 43 unique sequences in the CSF, which were parts of shared CSF/PB clusters compared with 990.3 (mean; range 110–1,749) in LGI1 antibody encephalitis. Shared clusters in the control were overwhelmingly PB dominated and had on average 1.2 CSF IgG sequences (range 1–3), whereas shared clusters in patients with LGI1 antibody encephalitis had larger CSF fractions with a mean of 8.1 CSF IgG sequences (range: mean value per patient 3.4–11.9; minimum/maximum value per patient across all 6 patients 1/465).

Taken together, DIRS demonstrated high sensitivity in detecting an intrathecal B-cell response and marked interconnectivity between the PB and CSF compartments in LGI1 antibody encephalitis, particularly of the more mature B-cell subsets. However, the antigen specificity of the clonally expanded intrathecal B cells remains unclear. Peripheral B-cell expansions infrequently reached the CSF, but those which did commonly lead to intrathecal B-cell expansions, consistent with secondary intrathecal B-cell receptor (BCR) diversification in LGI1 antibody encephalitis but not in noninflammatory controls.

## Discussion

Here, we examined the potential paradox between the limited detectable CSF activity in routine clinical assessments (e.g., CSF cell count and oligoclonal bands), alongside the presence of pathogenic CNS-active autoantibodies in patients with LGI1 antibody encephalitis. For the first time, using DIRS, we demonstrate highly active intrathecal B-cell activity in LGI1 antibody patients and show that B-cell repertoires, particularly from postgerminal center B cells on both sides of the BBB, may actively mutate and mature in patients with LGI1 antibody encephalitis.

Because, for methodological reasons, our study could not address the antigen specificity of the intrathecal B-cell response, it remains to be determined whether these expanded clones recognize LGI1-specific epitopes, and it is likely that some of these represent non–LGI1-specific B cells. LGI1-specific CSF B cells are more likely to exist in the patients in which intrathecal LGI1 antibodies were detectable. However, it cannot be excluded that LGI1 antibodies have passively diffused to the CSF.

In our studies, we found PB plasmablast/plasma cell IgG-VHs, which underwent SHM and were related to expanded clusters of CSF B cells. This pattern closely resembles findings in MS, suggesting that it may be a generic mechanism across CNS autoimmune conditions,^8,9,13^ and accordingly, we did not observe this phenomenon in noninflammatory controls. Yet, these observations appear different to the more limited intrathecal expansions observed in neuromyelitis optica spectrum disorder and the very low mutational load in CSF B cells from patients with NMDA receptor (NMDAR) antibody encephalitis.^14,15^ Hence, it may be that several different immunologic mechanisms operate across these autoantibody-mediated conditions. Our study did not aim to examine the antigen-specific population or correlate intrathecal B-cell responses with clinical outcome. Of interest, it has recently been observed that intrathecal LGI1 antibody synthesis correlates with a poorer prognosis.^16^ However, the lack of CSF LGI1 antibodies in some of our patients with striking intrathecal SHM may suggest that DIRS provides a more sensitive measure of B-cell activity.

In general, the majority of autoimmune encephalitis syndromes are considered to be IgG mediated.^2,7,16,17^ However, we also found evidence for extensively activated IgM-expressing peripheral B cells, which share similar or identical BCR heavy chains to intrathecal IgG-expressing B cells. The immunopathologic relevance of this IgM response is unknown, but may indicate an ongoing germinal center reaction-mediated stimulation of B cells as has been proposed in patients with NMDAR antibody encephalitis, where IgM NMDAR-reactive autoantibodies can be detected.^18,19^

A major question is whether interrupting these responses could potentially mitigate disease activity, prevent relapses, and improve long-term cognitive outcomes. Yet, only a subset of patients with LGI1 antibody encephalitis have been reported to respond favorably to rituximab, which targets—mainly peripheral—CD20-expressing B cells and has little effect on LGI1 antibody production.^20^ Our findings suggest that plasmablasts and plasma cells (which do not express CD20) are active in the CSF, and therefore, novel drugs that actively target these prolific protein synthesizers in the CNS compartment might be more effective than selective anti-CD20 treatments. Conversely, given that affinity maturation is an ongoing process in LGI1 antibody encephalitis and could result in increasingly efficient pathogenic antibodies, therapeutic depletion of CD20-expressing B-cell populations early in this disease might prevent development of chronic or progressive clinical phenotypes. Accordingly, early diagnosis of intrathecal immune activation in patients with mild clinical signs would be highly desirable.

## Glossary

BBB: blood-brain barrier
BCR: B-cell receptor
cDNA: complementary DNA
DIRS: deep B-cell immune repertoire sequencing
IgD: immunoglobulin D
IgG: immunoglobulin G
IgM: immunoglobulin M
LGI1: leucine-rich, glioma-inactivated 1
NMDAR: NMDA receptor
PB: peripheral blood
SHM: somatic hypermutation
SM: switched memory
UCSF: University of California, San Francisco
VH: heavy chain variable region
